# Amalgam Fillings

**Published:** 1876-03

**Authors:** J. C. Scott


### AMALGAM FILLINGS.

BY J. C. SCOTT, D. D. S.

The operation of filling a tooth with amalgam is one that should be performed with a great deal of care. In fact to fill a tooth with amalgam, and fill it perfectly, I regard as a difficult operation, not however because it is hard to press the *stuff* into a cavity, but for many other reasons which a man of experience knows.

I see every day one failure after another, and that too from the hands of the leading men in our profession. I have yet to see the first *perfect* amalgam filling, and my honest conviction is that a tooth can not be filled with the above mentioned material, but what will fail (if not at once), in the course of a few years. This does not apply however to all classes of teeth. I believe there are some teeth, those in which the tooth structure is firmly knit and the patient healthy, that can be filled (or rather pasted) with amalgam and last (as some of our worthy brothers tell their patients when speaking of teeth they had filled *twenty years ago* and are as good to-day as when first introduced) a score of years. But while this is true in one case out of ten thousand, the balance will be total failures. An instance here will fully illustrate what I have just said. A patient came to me some time since, wished his teeth examined and an approximation made to the probable cost. I examined his teeth and found some ten or twelve that needed attention, and that pretty badly; told him the fee for such an operation would be in the neighborhood of $75, or $80. This surprised him very much and he wished to know if there was not something cheaper they could be filled with and do just as well; that he had once heard of a man who had taken a shot and pressed it into a defective tooth and it never decayed again. He thought it would be much cheaper to have the teeth extracted and a plate inserted. It is of no use to dwell upon the arguments I used, for any fair dealing man knows just what I should have said to him, and did say.

He left my office and has just now turned up again with one of the hardest looking mouths I ever saw. In short it is a complete case of Ptyalism and most of the teeth in his mouth can be moved into almost any position. He is very sorry now that he did not take my advice, but instead went to Dr. ——— and let him put that filthy stuff into his teeth. I was so provoked when he came back to me, that I was almost tempted to send him away. My better feelings however prevailed, I pitied the man, and have him under treatment. I once heard Prof. J. Taft say in one of his lectures, that he had yet to put in his first amalgam filling; it has been ringing in my ears ever since and I have so often wished I could say the same thing.

I do not wish to be understood as saying that I do not fill teeth with amalgam, for I do, but the cases are few and far between. There is this about it though, I never use amalgam if I can in any way avoid it, and I never fill teeth with it that I know to be of frail structure, and consequently decay rapidly. No matter how thoroughly you pack the amalgam, while it is hardening and the mercury escaping, the filling must contract and by so doing may leave but the faintest line between the filling and periphery, but this is sufficient to give the same agents that produced decay in the first place a chance to go on with it again.

When I am obliged to fill a tooth with amalgam, the operation is performed in the ordinary way, except I apply the rubber dam as for a gold filling, having the cavity dry, using warm air that not a particle of moisture remains, then after the mercury is thrown off by the use of a chamois skin, the amalgam is placed on a small tin plate and warmed after which it is packed into the cavity as thoroughly as possible. Any one trying my method will find that warming the material is not a bad idea and is productive of good results. A filling thus introduced may last quite a number of years, but surely not a life time. The best plan is, never fill a tooth with amalgam when it can be avoided, and you will not hear so many complaints from your patients in regard to the failures you have made.

